# Influence of the Dose and Length of Wheat Fiber on the Quality of Model Sterilized Canned Meat Products

**DOI:** 10.3390/foods9081001

**Published:** 2020-07-26

**Authors:** Mirosław Słowiński, Joanna Miazek, Marta Chmiel

**Affiliations:** Division of Meat Technology, Department of Food Technology and Food Evaluation, Institute of Food Sciences, Warsaw University of Life Sciences-SGGW, 00-712 Warsaw, Poland; miroslaw_slowinski@sggw.edu.pl (M.S.); joanna-miazek@wp.pl (J.M.)

**Keywords:** wheat fiber, canned meat products, sterilization

## Abstract

The aim of this study was to evaluate the quality of model homogenized sterilized canned meat products produced with wheat fiber preparations (WF 200 R or WF 600 R) with different fiber lengths used in the amount of 3% or 6% by weight of the batter. Basic chemical composition (water, protein, fat, collagen and salt content), pH level, water activity, thermal drip, CIEL*a*b* color components, texture parameters (TPA, shear force) and sensory quality were determined. The addition of 3% or 6% of wheat fiber preparations did not affect the basic chemical composition, water activity and pH of products. The 6% addition of both fiber preparations caused lightening of the color of the meat blocks. Products with the addition of both wheat fiber preparations were characterized by significantly (*p* ≤ 0.05) higher hardness than the control product. Sensory quality of products, except tastiness, with the addition of wheat fiber preparations did not differ from the control product. There was no significant effect of wheat fiber length on the quality of meat blocks. Both lightening the color of canned meat blocks produced with the addition of wheat fiber preparation, as well as increasing their hardness, is desirable and contributes to increasing the quality of products.

## 1. Introduction

The purpose of fiber preparations in meat processing technology is relatively complex [1,2]. The studies carried out so far have shown that their inclusion in the technology of meat products is associated with the achievement of certain benefits, including: nutritional value enhancement, as fiber is a bioactive ingredient, the use of which enables the production of a meat products with a label containing a health or nutrition declaration; modification of composition and nutritional value such as fat substitution, energy reduction, replacement of some functional components marked as E-numbered ingredients (e.g., phosphates, emulsifiers, modified starch); increased binding and retention capacity of both water and fat, resulting in improved product yield and economy; control of water activity and extension of shelf-life; influence on texture parameters; reduction of diffusion and losses of volatile substances involved in the creation of taste and aroma sensations; emulsion stabilization; reduction of excessive drying and shrinkage of casings in dried products, and thus an improvement of their external appearance; neutral taste and aroma of most fiber preparations which do not adversely affect the sensory characteristics of the product [3,4,5,6,7,8,9,10,11]. With a sufficiently high fiber content in a meat product, it is possible to include nutrition or health declaration on the product’s label.

Currently, the market offers a wide range of fiber preparations (food cellulose, wheat, potato, oat, barley, apple, cranberry, chokeberry, flax, beet, bamboo, cocoa). They are produced from raw materials such as bran (wheat, oats, barley, corn and rice), supporting parts of plants (cereal husks and straw and corn cobs), root crops (potatoes, sugar beet pulp), legumes (peas, soya beans, beans) and wastes from the fruit and vegetable industry (e.g., waste from apples, carrots, citrus, tomatoes, chicory processing), etc. The characteristics of preparations are not only affected by the origin of the raw material, but also the production method [12,13,14,15,16,17,18]. The characteristics of the preparation are based on parameters such as the ability to bind water and oil, fiber length or granule size, solubility, bulk density or organoleptic characteristics such as color, aroma, taste [19,20]. 

The efficacy of fiber inclusion has been mostly determined in sausage or ham type meat products. There is no specific background and/or information in the literature on the application of fiber to sterilized canned meat products. Moreover, consumers are increasingly looking for food products with enhanced nutritional value [21]. Therefore, the aim of this work was to determine whether the use of high doses of fiber (authorizing the labeling of the product with a nutrition declaration related to the increased content of this ingredient, in accordance with applicable law) in the production of model homogenized sterilized canned meat products will be accepted in terms of technological and sensory quality of these products. In addition, it was determined whether this was also influenced by the length of this fiber. Because dietary fiber inclusion is rarely applied to canned meat products, this study is characterized by considerable novelty.

## 2. Materials and Methods 

### 2.1. Research Material

Model meat batters were produced from chilled (4 °C ± 1) chicken thigh meat, pork jowl and different additions of WF (Wheat Fiber) 200 R or WF 600 R Vitacel^®^ (Rettenmaier & Söhne GmbH + Co. KG/ Rosenberg, Germany) wheat fiber preparations (3% and 6%). According to the manufacturer’s data, the fiber preparations used in the study were characterized by dietary fiber content not lower than 97% in dry matter (including 94.5% of insoluble fraction). They differed in length and diameter of fibers. In the case of WF 200 R, it was 250 µm and 25 µm, respectively, and for WF 600 R, 80 µm and 20 µm, respectively.

### 2.2. Production of Model Homogenized Sterilized Canned Meat Products

Model meat batters were produced according to the following formula: chicken thigh meat (57.60%), pork jowl (10.20%), crushed ice (30.50%), curing mixture (99.4% of NaCl and 0.6% of NaNO_2_) (1.20%), sodium isoascorbate (0.04%), polyphosphate preparation (Tari P31; BK Giulini, Ladenburg, Germany) (0.23%) and spice mixture (ground black pepper and herb pepper in a 1:2 ratio; Kamis, Wólka Kosowska, Poland) (0.23%). Wheat fiber WF 200 R or WF 600 R preparations in the amount of 3 or 6% by weight of the model meat batters were also added. Five variants of model canned meat products were prepared: control variant, without addition of the wheat fiber preparation, variant with 3% addition of WF 200 R preparation, variant with 3% addition of WF 600 R preparation, variant with 6% addition of WF 200 R preparation and variant with 6% addition of WF 600 R preparation. 

The chicken thigh meat and pork jowl were ground in a laboratory grinder Mesko WN60 (Mesko AL. 2–4, MESKO-AGD Sp. z o.o., Skarżysko-Kamienna, Poland), equipped with a grid with holes of 3 mm diameter. Then the chopping process was carried out in the Hobart laboratory cutter (Hobart Manufacturing Company, Troy, OH, USA). The rotational speed of the knives was 1500 rpm. The temperature of the batters during the chopping process was monitored using a bayonet thermometer HI 98804 (Hanna Instruments, USA). The meat batters were prepared in three stages, and the following order of ingredient addition was maintained. In the first stage, chicken thigh meat, curing mixture and phosphate preparation were chopped for approximately 1 min. In the second stage crushed ice, sodium isoascorbate and fiber preparations (in appropriate variants) were added and chopping time was approximately 3 min. In the third stage, the pork jowl and spices were added and the chopping time was approximately 6 min until the final temperature of the meat batter reached the level of max 12 °C.

Metal cans with a diameter of 76 mm and a height of 54 mm were filled with the model meat batters by introducing 190 g (±0.1 g) of batter into each can. For each product variant, 10 cans were prepared. After filling, the packages were vented and then closed with a “double overlap” using a semi-automatic closing machine (Nov-Handy Novopacké, Wahlstedt, Germany). Model meat batters were left for approximately 6 h to cure under refrigerated conditions (temperature 4 °C ± 1). After this time, the cans with meat batters were randomly placed inside a water-steam autoclave (A-125E, Jugema, Środa Wielkopolska, Poland) initially pre-heated to 95 °C and the sterilization process itself was carried out at 121.1 °C until the commercial sterilization value of F_0_ ≥ 3.0 was reached in the geometric center of the product. From that moment the cooling process in the autoclave was started. To control the parameters of the sterilization process in each production series, in one can a hole was made in the middle of its height and a special feedthrough was placed inside, enabling installation of sensors (TrackSense^®^ PRO Logger, Ellab, Hilleroed, Denmark). The sensors recorded the temperature inside the autoclave and in the geometric center of the can every 30 s, with an accuracy of 0.1 °C. When it was possible to remove the canned products from the autoclave after sterilization, the further cooling process was carried out in a water-ice bath. After, the cans were removed from the water-ice bath, were dried with a paper towel, placed in a cooling room (temperature 4 °C ± 1) and stored for 24 h. After this time, the evaluation of model canned meat products quality was carried out and included the assessment of the content of basic chemical components (water, protein, fat, collagen and salt), pH level, water activity, thermal drip, L*, a* and b* color components, texture parameters (TPA – Texture Profile Analysis, shear force) and sensory quality. The experiment was carried out three times in three independent production series.

### 2.3. Methods

#### 2.3.1. Content of the Basic Chemical Components

Determination of water, protein, fat and collagen content was done by the method of near-infrared reflectance transmission (NIT) using calibration on artificial neural networks (ANN) in a FoodScan apparatus (FOSS Analytical, Warsaw, Poland) and according to PN-A-82109 [22]. Each, previously minced, sample of finished product was evenly placed in a glass circular scale inside the apparatus and the measurement was made. The salt content was determined using potentiometric methods according to PN-ISO 1841–2:2002 [23], using a 702 SM Titrino apparatus (Metrohm AG, Herisau, Switzerland) device. The analyses were determined on 3 samples taken from 3 different cans for each variant of the product in each of three production series and the mean value was taken as a result.

#### 2.3.2. pH Measurement

The pH level of the model homogenized sterilized canned meat products was evaluated using a CP-401 pH-meter (Elmetron, Zabrze, Poland) calibrated with pH 4 and pH 7 buffers. Prior to taking the pH measurement, 10 g of previously minced finished product and 20 cm^3^ of distilled water were placed in a 50 cm^3^ glass beaker; next, all the ingredients were thoroughly mixed and then left for 10 min. A combined glass-calomel electrode and temperature compensation sensor were inserted into the analyzed solutions. The pH level of the product was determined on 3 samples taken from 3 different cans for each variant of the product in each of three production series and the mean value was taken as a result.

#### 2.3.3. Water Activity Measurement

Measurement of water activity was carried out using an Aqua Lab CX-2 apparatus (Decagon Devices, Inc., Pullman, WA, USA). The samples were prepared by cutting a flat rectangle from the center of the meat block. The analysis was performed at a temperature of 20.0 ± 1.0 °C. The water activity of the product was determined on 3 samples taken from 3 different cans for each variant of the product in each of three production series and the mean value was taken as a result.

#### 2.3.4. Thermal Drip Determination

Determination of the amount of leakage after heat treatment (thermal drip) was made according to PN-85/A-82056 [24]. The model canned meat products were opened from both sides and then the meat block was pushed out and purified, via scraping with a knife, from jelly and then weighed. The result was expressed as a percentage in relation to the net weight of the canned meat batter. The thermal drip of the product was determined on 3 different cans for each variant of the product in each of three production series and the mean value was taken as a result.

#### 2.3.5. Color Measurement

Color parameters were measured in the CIEL*a*b* scale (Comission Internationale de l’Eclairage) [25] at the freshly cut canned meat block using a Minolta CR200 colorimeter (Konica Minolta, Wroclaw, Poland; light source D_65_, observer angle 10, measuring head hole 8 mm) calibrated according to white standard (L* 99.18, a*−0.07, b*−0.05). For each variant of the product, in each production series, each measurement was performed 5 times on 3 samples taken from 3 different cans and the mean value was used as the final result.

#### 2.3.6. Texture Parameters Measurement

Measurement of texture parameters was performed 24 h after the production using a Zwicki 1120 (Zwick GmbH&Co., Ulm, Germany) texture analyzer. The samples (in cans before opening) were conditioned at 20 °C for 4 h to align the temperature. Texture Profile Analysis (TPA) was performed using a flat adapter. The samples for the study were the cylinders with a diameter of 10 mm and a height of 20 mm cut from a meat products. Samples were double-compressed between two parallel sheets of the apparatus. The head speed was 50 mm/min during the analysis. During the first cycle of compression, the sample was 30% deformed. Once initiated, the second cycle continued until this value decreased upon the sample deformation. A testExpert software (v.12.0, 2006, Zwick GmbH & Co., Ulm, Germany) was used to calculate the values of the following parameters: cohesiveness, springiness, hardness and chewiness. The parameters were determined as follows: cohesiveness, ratio between the work performed during the second compression and the work performed during the first compression; springiness, ratio between the second peak base and the first peak base, hardness, maximum force value [N] at a first sample compression; chewiness, product of firmness, cohesiveness, and springiness. The analysis was determined on 3 samples taken from 3 different cans for each variant of the product in each of three production series and the mean value was taken as a result.

Shear force measurement was made on cubes of 20 mm × 20 mm × 40 mm dimension cut from product. Shear force measurement was performed with the use of Warner–Bratzler adapter equipped with V-blade knife. The maximum force needed to cut the samples, at a speed of movement of the measuring head of 50 mm/min with an initial force of 0.5 N, was measured. The analysis was determined on 3 samples taken from 3 different cans for each variant of the product in each of three production series and the mean value was taken as a result.

#### 2.3.7. Sensory Evaluation

Sensory evaluation was carried out by a team composed of 15 students and employees of Division of Meat Technology WULS-SGGW, trained in terms of the evaluated parameters. The samples of model canned meat products (in cans before opening) were evaluated after conditioning at 20 °C for 2 h. As samples for sensory evaluation, slices were used which had been cut from a block of meat with a thickness of about 2 cm. The sensory panel used a 10 point scale, where 0 meant no acceptance at all and 10 meant the highest level of product acceptance, as described by Baryłko-Pikielna and Matuszewska (2009) [26]. The general appearance, structure and consistency, tastiness, color, odor and general desirability were evaluated. For sensory evaluation, the products from 4 cans of each variant in each production series were used. Based on individual results for each descriptor, the average values were determined.

#### 2.3.8. Statistical Analysis

The obtained results were subjected to statistical analysis using Statistica ver. 12 PL software (StatSoft, Inc., Tulsa, OK, USA). To determine the significance of differences between average values of quality characteristics of model homogenized sterilized canned meat products, a one-way analysis of variance (one-way ANOVA) was used. Significant differences between treatments were verified on the basis of Tukey’s HSD test, at a significance level of α = 0.05.

## 3. Results and Discussion

Discussion of the results obtained in this study is difficult because there are no studies on the use of fiber in sterilized meat products. Therefore, even though our model canned meat product is treated by sterilization process (unlike other meat products) we decided to compare results with sausage or ham type meat products prepared with dietary fiber.

The conducted study did not show any significant (*p* > 0.05) influence for the addition of both wheat fiber preparations on the basic chemical composition, collagen and salt content in model canned meat products (Table 1). 

This results from, inter alia, the fact that the used formula guaranteed very good water retention by the batter (despite the relatively high water content), and as a result, there was no leakage in the can. However, depending on the fiber type and/or meat product the protein content may decrease with an increase in fiber concentration [9,27]. According to Cegiełka et al. [28], the enrichment of ready-to-eat meat products (chicken burgers) with wheat fiber did not differentiate their chemical composition when compared to products prepared without the fiber.

Regardless of the dose and length of the fiber, there was no influence (*p* > 0.05) of the used fiber preparations on the pH of the model homogenized sterilized canned meat products (Table 2). The obtained results correlate with the reports [1,16] confirming the lack of influence of wheat fiber preparations on the pH of meat batters and are also consistent with the declaration included in the specifications of the preparations used in our study.

It is usually assumed that dietary fiber preparations are a factor in reducing the water activity of food [27,29]. However, in our studies, no influence (*p* > 0.05) of the addition of the fiber preparations on reduction of water activity was found (Table 2).

Taking into account the formula composition, it can be assumed that it resulted from the amount of water added to the batters. Moreover, the use of multi-phosphate preparation additionally contributed to the increase of water binding capacity of proteins in the model system. The relatively high content of muscle proteins of poultry meat also favored the binding of water and these components could compete with the wheat fibers. It could be due to the order in which the components were added during the batter production process. In each of the variants, including control one, muscle proteins probably bound water added to the system in the first place, so that the added fiber preparation could only serve as a stabilizer of the produced batters. In each of the analyzed variants no thermal drip was found (Table 2).

With the increase in the addition of both (WF 200 R, WF 600 R) fiber preparations from 3% to 6%, the color of canned meat blocks was lightened (Table 2). Values of the L* color component were significantly (*p* ≤ 0.05) higher for model meat batters with 6% addition of fiber preparations, compared to a control product or a product with 3% additive of fiber preparations. The addition of both preparations of wheat fiber caused a slight decrease in the a* color component values (redness) and a significant (*p* ≤ 0.05) increase in the b* color component (yellowness) in comparison to the control variant (Table 2). These differences increased proportionally to the dose of the applied additive (from 3 to 6%) within the same fiber preparation (Table 2). Tendencies in the color change of the model homogenized sterilized canned meat products were influenced to a greater extent by the preparation with longer fibers. According to Choi et al. [30,31], addition of dietary fibers decreased the redness of meat products during cold storage. 

The results obtained for cohesiveness, describing the degree of binding of the studied material, showed that the addition of wheat fiber preparations did not cause a significant change in its value (Table 3). Differently, Barretto et al. [1] showed that the wheat fiber addition significantly contributed to the decrease of the cohesiveness of bologna sausages. The addition of both fiber preparations, regardless of the applied dose, did not significantly (*p* > 0.05) affect the springiness of canned meat blocks (Table 3). This parameter is related to the distortion as a result of deformation and the degree of return of the deformed material to the state before deformation, thus indicating the elasticity and plasticity of the obtained canned meat blocks. 

Taking into account the results obtained for the parameter defined as hardness of the products, it was found that the homogenized sterilized canned meat products made with the addition of 6% of both wheat fiber preparations (WF 200 R or WF 600 R) had significantly (*p* ≤ 0.05) higher hardness compared to the control variant (Table 3). A greater hardness of the product upon greater quantity of added wheat fiber was also observed by Barretto et al. [1]. Comparing the values of chewiness, only a slight, but insignificant *(p* > 0.05) increase in the value for this parameter with an increase in the addition (from 3% to 6%) of both fiber preparations was observed (Table 3).

No significant (*p* > 0.05) effect of addition of both preparations of wheat fiber on the shear force values in comparison with the control variant was found (Table 3). A tendency was only noted, according to which the shear force values were slightly higher in the case of canned meat products containing longer fiber preparations (WF 200 R), in comparison with the variants with shorter fibers (WF 600 R). The reason for the lack of differences in most texture parameters was probably a high addition of water provided in the recipe of the products and the sterilization process.

The addition of both preparations of wheat fiber (WF 200 R or WF 600 R) did not significantly (*p* > 0.05) affect the deterioration of the perception of most of the sensory evaluated parameters of the model homogenized sterilized canned meat products, and in some cases contributed to an increase in the mean scores awarded during the evaluation (Table 4).

In the opinion of the panelists, in most of the evaluated parameters, products with the addition of preparations with longer fibers (WF 200 R) were better evaluated in relation to products produced with preparations with shorter fibers (WF 600 R). The only lesser rated sensory parameter was consistency, which was described as more compact (Table 4). What is more important, i.e., the tastiness, the model homogenized sterilized canned meat product with 6% addition of fiber preparation with shorter fibers (WF 600 R) was evaluated significantly (*p* ≤ 0.05) lower than the control variant and product with 3% addition of WF200 R fiber preparation. According to Eim et al. [12], the meat products with the addition of carrot fiber presented a high overall acceptability, over 75% for all attributes, achieving higher scores than the samples without fiber.

In conclusion, it was found that in the case of model homogenized sterilized canned meat products, wheat fiber length does not affect the quality of product. It was possible to produce sensory acceptable canned meat products containing 6% wheat fiber, which would allow for the declaration "high fiber content".

## 4. Conclusions

The addition (3% or 6%) of wheat fiber preparations with different length of fibers (WF 200 R or WF 600 R) does not affect the basic chemical composition (water, protein, fat, collagen and salt content), water activity and pH of the model homogenized sterilized canned meat products, which indicates the lack of influence of the length of wheat fiber on the quality of sterilized, homogenized canned meat product. The applied 6% addition of wheat fiber preparations WF 200 R, WF 600 R caused lightening of the color of canned blocks. The addition of both tested preparations of wheat fiber caused an increase in the hardness of canned meat blocks in comparison to the control product. Significantly higher values of this parameter were observed in most cases for variants containing higher (6%) addition of fiber. This indicates the possibility of modification and shaping of texture parameters of model homogenized sterilized canned meat product due to the use of fiber preparations. The sensory evaluation showed that the incorporation of wheat fiber preparations WF 200 R or WF 600 R in the model homogenized sterilized canned meat products could be made, in most cases, without interfering in the acceptance of the product. It was possible to produce sensory and technologically acceptable canned meat products containing 6% of wheat fiber, which would allow for the declaration "high fiber content".

## Figures and Tables

**Table 1:** Basic chemical composition of the model homogenized sterilized canned meat products (mean ± standard deviation)**.**

Content [%]	Product Variant				
	Control	WF200 R 3%	WF600 R 3%	WF200 R 6%	WF600 R 6%
water	73.8 ± 0.8	73.5 ± 0.8	74.0 ± 0.1	73.4 ± 1.3	73.0 ± 1.0
protein	13.1 ± 0.3	13.0 ± 0.2	12.6 ± 0.5	12.4 ± 0.2	12.6 ± 0.2
fat	9.3 ± 1.1	9.3 ± 0.8	9.0 ± 0.7	10.0 ± 1.1	9.3 ± 1.1
collagen	1.6 ± 0.4	1.5 ± 0.3	1.7 ± 0.3	1.5 ± 0.4	1.6 ± 0.4
salt	1.9 ± 0.1	1.9 ± 0.1	2.0 ± 0.1	1.9 ± 0.1	1.9 ± 0.1

WF200 R – wheat fiber with fibers of 250 µm length and 25 µm diameter. WF 600 R – wheat fiber with fibers of 80 µm length and 20 µm diameter.

**Table 2:** Selected quality characteristics of the model homogenized sterilized canned meat products (mean ± standard deviation).

Characteristic	Product Variant				
	Control	WF200 R 3%	WF600 R 3%	WF200 R 6%	WF600 R 6%
pH	6.6 ± 0.1	6.6 ± 0.1	6.6 ± 0.1	6.6 ± 0.1	6.6 ± 0.1
water activity	0.975 ± 0.002	0.975 ± 0.002	0.973 ± 0.001	0.973 ± 0.001	0.972 ± 0.002
thermal drip [%]	No drip loss				
L*	71.73 ^a^ ± 0.62	71.72 ^a^ ± 0.15	71.13 ^a^ ± 0.71	73.09 ^b^ ± 0.69	72.97 ^b^ ± 0.71
a*	7.05 ± 0.64	6.99 ± 0.45	6.76 ± 0.72	6.08 ± 0.66	6.32 ± 0.59
b*	8.09 ^a^ ± 0.01	9.21 ^b^ ± 0.41	9.17 ^b^ ± 0.57	10.10 ^b^ ± 0.34	9.74 ^b^ ± 0.49

^a,b^ - means in the row marked with different letters are significantly different (*p* ≤ 0.05). L*, a*, b* - color components: lightness, redness, yellowness.

**Table 3:** Texture parameters of the model homogenized sterilized canned meat products (mean ± standard deviation).

Characteristic	Product Variant				
	Control	WF200 R 3%	WF600 R 3%	WF200 R 6%	WF600 R 6%
cohesiveness	0.58 ± 0.03	0.54 ± 0.01	0.55 ± 0.03	0.53 ± 0.00	0.56 ± 0.02
springiness	0.73 ± 0.04	0.73 ± 0.03	0.73 ± 0.02	0.72 ± 0.02	0.72 ± 0.02
hardness (N)	9.57 ^a^ ± 1.00	11.08 ^a,b^ ± 1.03	10.63 ^a,b^ ± 0.46	12.94 ^b^ ± 0.29	12.41 ^b^ ± 0.72
chewiness (N)	4.75 ± 0.71	4.41 ± 0.97	4.28 ± 0.89	4.95 ± 0.63	5.10 ± 0.92
shear force (N/cm^2^)	0.55± 0.06	0.60 ± 0.10	0.56 ± 0.12	0.80 ± 0.17	0.69 ± 0.15

^a,b^ - means in the row marked with different letters are significantly different (*p* ≤ 0.05).

**Table 4:** Sensory quality of the model homogenized sterilized canned meat products (mean ± standard deviation).

Characteristic (Points)	Product Variant				
	Control	WF200 R 3%	WF600 R 3%	WF200 R 6%	WF600 R 6%
general appearance	6.0 ± 1.3	6.1 ± 1.0	5.9 ± 1.0	5.6 ± 1.3	5.5 ± 1.1
structure and consistency	4.8 ± 0.8	4.6 ± 0.5	4.3 ± 0.9	4.9 ± 1.2	4.5 ± 1.2
tastiness	5.3 ^a^ ± 0.9	5.6 ^a^ ± 0.7	4.8 ^a,b^ ± 1.1	4.0 ^a,b^ ± 0.7	3.6 ^b^ ± 0.3
color	6.3 ± 0.7	6.8 ± 0.5	6.6 ± 0.5	5.5 ± 1.0	5.1 ± 1.0
odor	5.3 ± 0.9	5.3 ± 0.7	5.7 ± 0.5	5.3 ± 0.8	5.8 ± 0.5
general desirability	5.2 ± 1.0	5.4 ± 0.8	5.1 ± 0.8	4.9 ± 0.8	4.7 ± 1.0

^a,b^ - means in the row marked with different letters are significantly different (*p* ≤ 0.05).
